# Resistance beyond the plate: heavy metal, disinfectant, and antibiotic resistance in foodborne *Staphylococcus aureus* isolates

**DOI:** 10.1093/femsle/fnaf109

**Published:** 2025-10-06

**Authors:** Bahar Onaran Acar, Hakan Şenarısoy, Hidayet Bozdoğan

**Affiliations:** Department of Food Hygiene and Technology, Faculty of Veterinary Medicine, Ankara University, Ankara, 06110, Türkiye; Sütas Dairy Group, Research and Development Center, Bursa, 16700, Türkiye; Ministry of Agriculture and Forestry, General Directorate of Food and Control, Ankara, 06800, Türkiye

**Keywords:** resistance, heavy metal, disinfectants, antibiotic, foodborne, *Staphylococcus aureus*

## Abstract

*Staphylococcus aureus* is a major foodborne pathogen capable of developing resistance to multiple antimicrobial agents, including antibiotics, disinfectants, and heavy metals. This study investigated 63 *S. aureus* isolates recovered from poultry products, meat cuts, and processed or ready-to-eat foods for their resistance to selected disinfectants [benzalkonium chloride (BC), cetylpyridinium chloride (CPC), triclosan (TCS)] and heavy metals [zinc (Zn), nickel (Ni), mercury (Hg), copper (Cu), cadmium (Cd), lead (Pb), cobalt (Co)]. The isolates from processed and ready-to-eat foods exhibited significantly higher resistance scores to antibiotics, disinfectants, and heavy metals compared to those from meat and poultry (*P* < .001). Notably, 45% of isolates from processed foods were resistant to 8 out of 10 tested agents, and enterotoxigenic isolates displayed significantly elevated resistance profiles across all tested agent classes (antibiotics, heavy metals, disinfectants) (*U* = 99.0, *P* < .001). These findings highlight the potential role of food processing environments in selecting for multidrug-resistant and virulent *S. aureus* strains. The co-occurrence of resistance to antibiotics, disinfectants, and heavy metals suggests the involvement of co-selection mechanisms, emphasizing the need for integrated surveillance, and targeted interventions to reduce the spread of resistant pathogens in the food chain.

## Teaser

*Staphylococcus aureus* isolates without enterotoxin genes tend to show higher resistance to antibiotics and heavy metals, highlighting a potential trade-off between virulence and antimicrobial resistance.

## Introduction

*Staphylococcus aureus* is a significant foodborne pathogen, widely recognized for its ability to develop resistance to multiple antibiotics, disinfectants, and environmental stressors such as heavy metals. In addition to its well-documented antibiotic resistance, the increasing prevalence of *S. aureus* strains resistant to disinfectants—particularly quaternary ammonium compounds and phenolic agents—and toxic metals poses additional challenges for food hygiene, safety, and public health. These adaptive traits not only improve bacterial survival in hostile environments but may also contribute to the co-selection and persistence of antimicrobial resistance (Chieffi et al. 2023).

Co-selection refers to the phenomenon where exposure to one selective agent, such as a disinfectant or heavy metal, simultaneously promotes resistance to unrelated agents like antibiotics. This occurs when genes conferring resistance to multiple stressors are physically linked on the same mobile genetic elements, such as plasmids or transposons (Wales and Davies 2015, Li et al. 2022). In food production settings, this co-selection effect can severely compromise hygiene strategies, allowing resistant bacteria to persist despite routine sanitization measures.

Heavy metals—including zinc (Zn), nickel (Ni), mercury (Hg), copper (Cu), cadmium (Cd), lead (Pb), and cobalt (Co)—are environmental contaminants that may enter the food chain through feed additives, processing equipment, water, and packaging materials. Their accumulation in food environments exerts selective pressure on microbial communities, encouraging the survival of resistant strains. Likewise, the extensive use of disinfectants such as benzalkonium chloride (BC), cetylpyridinium chloride (CPC), and (TCS) in food processing facilities can lead to the emergence and persistence of disinfectant-resistant *S. aureus* strains (Wales and Davies 2015, Cufaoglu et al. 2022).

Recent studies have increasingly documented the co-occurrence of resistance to biocides, antibiotics, and heavy metals in *S. aureus*, particularly among isolates from foods of animal origin (Li et al. 2022, Onaran et al. 2025). These findings suggest that cross-resistance and co-selection mechanisms may be widespread, especially in high-risk food categories such as poultry products, meat cuts, processed foods, and ready-to-eat items (Chieffi et al. 2023). Such products often undergo complex processing and are subject to repeated exposure to various sanitizing and preservation agents, potentially facilitating the development and dissemination of resistant strains.

The aim of this study is to investigate the distribution and co-occurrence of antibiotic, disinfectant, and heavy metal resistance traits among *S. aureus* isolates obtained from diverse food categories. Additionally, the toxigenic profiles of these isolates were evaluated to explore potential associations between virulence and resistance traits. By characterizing resistance patterns across food types and assessing the role of enterotoxin gene carriage, this research contributes valuable data to foodborne pathogen surveillance, antimicrobial resistance risk assessment, and the understanding of co-selection dynamics in food-associated *S. aureus* populations.

## Materials and methods

### Bacterial strains

A total of 63 *S. aureus* strains were included in this study. These isolates had been previously obtained from various food products—including poultry meat, meat cuts, processed foods, and ready-to-eat dairy-based desserts—and were previously characterized in terms of their antibiotic resistance profiles and staphylococcal enterotoxin (SE) genes. In the present study, these same isolates were further analyzed to evaluate their resistance to selected heavy metals and disinfectants. The standard strain of *S. aureus* ATCC 25923 was used on a positive control well (Yang et al. 2020). "Supplementary material" presents the previously determined antibiotic resistance profiles and SE gene carriage of the isolates.

### Determination of heavy metal MIC values

The minimum inhibitory concentration (MIC) of various heavy metals was determined using the broth microdilution method, in accordance with the guidelines set by the Clinical and Laboratory Standards Institute (CLSI 2015). The heavy metals tested included cobalt (CoCl_2_), zinc (ZnCl_2_), cadmium (CdCl_2_), copper (CuCl_2_·2H_2_O), mercury (HgCl_2_), nickel (NiCl_2_), and lead (PbCl_2_). Stock solutions were prepared by dissolving appropriate heavy metal salts in distilled water, which were then sterilized using a 0.22 µm Millipore filter. The concentration range for the metal solutions was maintained between 6.25 and 3200 µg/ml for all metals, except Hg, which was tested in the range of 0.78–400 µg/ml (He et al. 2016, Dahanayake et al. 2019).

Bacterial isolates were first cultured overnight in Mueller-Hinton Broth and incubated at 37°C. Prior to testing, the bacterial suspensions were standardized to a 0.5 McFarland turbidity, yielding a final inoculum of 5 × 10^5^ CFU/ml, as determined using a Biosan Den-1B McFarland Densitometer (ISO 20776–1, 2019). Each well of a 96-well microtiter plate was inoculated with 50 µl of the diluted heavy metal solutions and 50 µl of bacterial suspension. The plates were then sealed with a transparent film and incubated at 37°C for 24 h. Following incubation, proper growth in the positive control wells and no growth in the negative control wells were confirmed. The MIC value was defined as the lowest concentration of metal that completely inhibited bacterial growth (Wu et al. 2015). The experiment was carried out in triplicate, and any isolate with MIC values exceeding those of the standard strains was considered resistant.

### Determination of disinfectant MIC values

Resistance to disinfectants, including BC, CPC, and TCS, was evaluated using the broth microdilution method, following the CLSI guidelines (CLSI 2015). The disinfectant concentrations for the MIC determination ranged from 0.125 to 1024 mg/l, as previously outlined in studies by Zou et al. (2014) and Wu et al. (2015). In this procedure, 50 µl of each disinfectant solution at varying concentrations and 50 µl of the bacterial suspension were added to the respective wells of a 96-well microtiter plate. The plates were sealed and incubated at 37°C for 24 h. Following incubation, growth was checked in both the positive control and negative control wells to ensure proper conditions. The MIC value for each disinfectant was recorded as the lowest concentration at which no bacterial growth was observed. The experiment was conducted in triplicate, and the resistance profiles of the isolates were compared with the control strains—isolates displaying MIC values greater than those of the standard strains were classified as resistant.

### Statistical analysis

All statistical analyses were conducted using IBM SPSS Statistics version 21.0. To assess whether resistance levels to antibiotics, disinfectants, and heavy metals differed significantly among the three food source groups (Poultry Products, Meat Cuts, and Processed/Ready-to-eat Foods), a one-way analysis of variance (ANOVA) was employed. Prior to analysis, assumptions of normality and homogeneity of variances were evaluated. When the ANOVA yielded statistically significant results, post-hoc multiple comparisons were performed using Tukey’s Honestly Significant Difference (HSD) test to identify pairwise differences between groups. A two-tailed *P*-value of < .05 was considered statistically significant.

To evaluate the potential impact of enterotoxin gene presence on resistance levels, isolates were stratified into two groups: enterotoxigenic and nonenterotoxigenic. Resistance scores to antibiotics, disinfectants, and heavy metals were compared between these two groups using the nonparametric Mann–Whitney U test, due to nonnormal data distribution. A *P*-value less than .05 was considered statistically significant. All analyses were conducted using IBM SPSS Statistics version 21.0.

## Results

A total of *Staphylococcus aureus* isolates were obtained from various food categories, including poultry products, meat cuts, and processed or ready-to-eat foods. These isolates were evaluated for resistance to selected disinfectants (BC, CPC, TCS) and heavy metals (Zn, Ni, Hg, Cu, Cd, Pb, Co), and the potential co-selection between these resistance traits was assessed.

The isolates obtained from poultry products showed variable resistance: 81.8% (18/22) to BC, 40.9% (9/22) to CPC and Pb, 90.9% (20/22) to Zn and Hg, 54.5% (12/22) to Cu, 18.2% (4/22) to Cd, 63.6% (14/22) to Co, and 9.1% (2/22) to TCS. In contrast, isolates from meat cuts were resistant to 42.8% (9/21) BC, 23.8% (5/21) CPC, 90.5% (19/21) Zn, 85.7% (18/21) Hg, 61.9% (13/21) Cu, 28.6% (6/21) to Cd, 33.3% (7/21) Pb, and 52.4% (11/21) Co, but none showed resistance to TCS and Ni. Among isolates from processed and ready-to-eat products, resistance was detected in 40% (8/20) for CPC, 55% (11/20) for BC and Pb, 90% (18/20) for Zn, 75% (15/20) for Hg, and 70% (14/20) for Cu, Cd, and Co. No resistance to TCS and Ni was observed in any category. Importantly, 45% (9/20) of isolates from processed and ready-to-eat products exhibited resistance to 8 out of the 10 evaluated agents, compared to only 4.5% from poultry products. These isolates also displayed a broader antibiotic resistance profile relative to other food categories.

The isolates displayed varying resistance profiles. Resistance to heavy metals such as Zn and Hg was the most commonly observed, particularly among isolates from processed and ready-to-eat foods. Among disinfectants, resistance to BC was more prevalent compared to CPC and TCS. Several isolates exhibited multi-resistance to both disinfectants and heavy metals. Notably, enterotoxin genes were detected in isolates with and without resistance traits (Supplementary Material).

One-way ANOVA analysis revealed statistically significant differences in resistance levels to antibiotics (*P* < .001), disinfectants (*P* < .001), and heavy metals (*P* < .001) among the sample groups (Poultry Products, Meat Cuts, and Processed/Ready-to-eat Foods). Post-hoc Tukey’s HSD test indicated that isolates from the Processed and Ready-to-eat Foods group exhibited significantly higher resistance scores when compared to those from Meat Cuts and Poultry Products. Specifically, isolates recovered from processed and ready-to-eat foods exhibited significantly higher mean resistance levels compared to other groups: for antibiotics, the mean difference was 4.1 (*P* < .001) compared to Meat Cuts and 3.7 (*P* < .001) compared to Poultry Products; for disinfectants, the differences were 4.3 (*P* < .001) and 4.1 (*P* < .001), respectively; and for heavy metals, the mean resistance was 4.0 units higher than in isolates from Meat Cuts (*P* < .001). These findings suggest a potential correlation between the level of food processing and increased microbial resistance, possibly due to selective pressure from environmental exposure to antimicrobials and heavy metals throughout processing and handling stages.

In Table 1, Tukey’s HSD test results are presented, showing significant pairwise differences in resistance levels to antibiotics, disinfectants, and heavy metals among food sample groups. Only comparisons with statistically significant differences (*P* < .05) are included. The table demonstrates that isolates from Processed and Ready-to-eat Foods exhibit significantly higher resistance to these agents compared to those from Meat Cuts and Poultry Products, suggesting a strong association between food processing and elevated resistance profiles.

**Table 1.** Tukey’s HSD analysis revealing elevated resistance in processed food isolates

Group 1	Group 2	Mean difference	*P*-adj	Lower	Upper	Reject	Resistance type
Meat	Processed	4.1	< .001	3.1698	5.0302	True	Antibiotic
Poultry	Processed	3.7	<.001	2.7698	4.6302	True	Antibiotic
Meat	Processed	4.3	<.001	3.5455	5.0545	True	Disinfectant
Poultry	Processed	4.1	<.001	3.3455	4.8545	True	Disinfectant
Meat	Processed	4.0	<.001	3.3563	4.6437	True	Heavy metal
Poultry	Processed	4.2	<.001	3.5563	4.8437	True	Heavy metal

Comparison of resistance profiles between enterotoxigenic and nonenterotoxigenic *S. aureus* isolates revealed statistically significant differences across all tested categories (Table 2). Enterotoxigenic isolates demonstrated higher resistance levels to antibiotics, disinfectants, and heavy metals when compared to their nontoxigenic counterparts. Specifically, Mann–Whitney U test results indicated that resistance scores were significantly greater among enterotoxigenic isolates for antibiotics (*U* = 99.0, *P* < .001), disinfectants (*U* = 99.0, *P* < .001), and heavy metals (*U* = 99.0, *P* < .001). These findings suggest a potential association between virulence and resistance traits, indicating that toxin-producing strains may also possess increased tolerance to various antimicrobial and environmental stressors.

**Table 2.** Resistance to disinfectants and heavy metals by enterotoxigenicity

Enterotoxigenicity	Total Isolates	BC	CPC	TCS	Zn	Hg	Cu	Cd	Pb	Co	Ni
Yes	9	4/9 (44.4%)	2/9 (22.2%)	0/9 (0.0%)	9/9 (100.0%)	8/9 (88.9%)	5/9 (55.6%)	3/9 (33.3%)	4/9 (44.4%)	7/9 (77.8%)	0/9 (0.0%)
No	54	34/54 (63.0%)	20/54 (37.0%)	0/54 (0.0%)	48/54 (88.9%)	45/54 (83.3%)	33/54 (61.1%)	23/54 (42.6%)	24/54 (44.4%)	32/54 (59.3%)	0/54 (0.0%)

**Figure 1.**
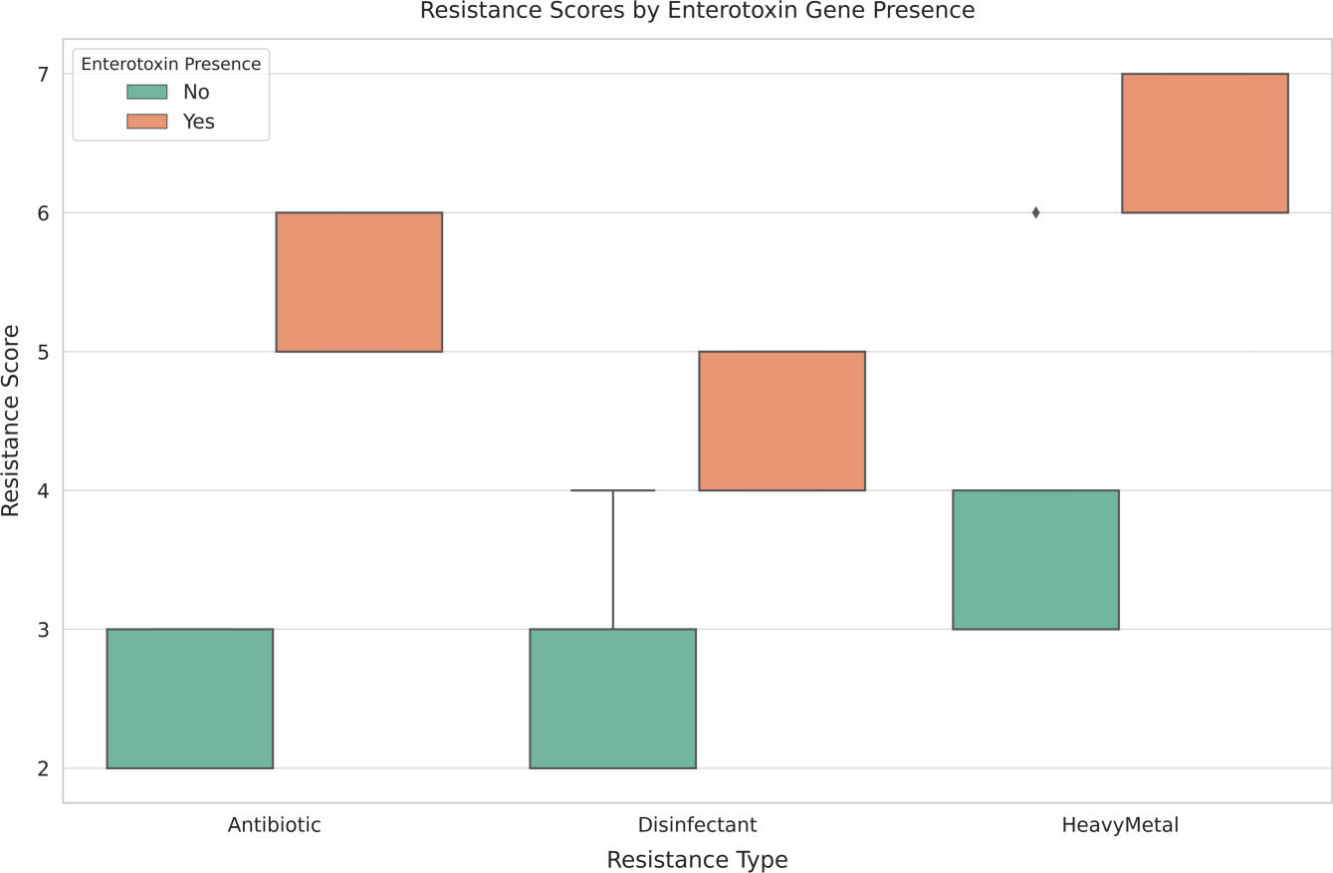
Comparison of cumulative resistance scores among enterotoxigenic (*n* = 9) and nonenterotoxigenic (*n* = 54) *Staphylococcus aureus* isolates. Resistance score was calculated as the sum of agents (antibiotics, disinfectants, heavy metals) to which each isolate was resistant. Data are presented as mean ± standard deviation. Statistical significance was determined using the Mann–Whitney U test (*P* < .05).

Fig. 1 illustrates that *S. aureus* isolates carrying enterotoxin genes exhibit consistently higher resistance scores across all tested categories—antibiotics, disinfectants, and heavy metals—compared to nontoxigenic isolates. The elevated median values and narrower interquartile ranges in the enterotoxigenic group suggest a stronger and more uniform resistance profile, supporting the hypothesis that virulence and resistance traits may co-occur due to shared selective pressures or genetic linkage.

## Discussion

The detection of *Staphylococcus aureus* strains resistant to disinfectants, heavy metals, and multiple antibiotics in food products, especially processed and ready-to-eat items, poses a serious concern for both food safety and public health. The high frequency of resistance to Zn and Hg may reflect the widespread use of these compounds in food production environments (Leguillier et al. 2024). Although the number of isolates from certain categories was limited, the resistance patterns observed provide meaningful trends that warrant further investigation. These findings underscore the need for continuous surveillance and targeted mitigation strategies.

Isolates from ready-to-eat food products exhibited significantly higher resistance to both disinfectants and heavy metals compared to those from poultry and meat. This may be attributed to the selective pressure exerted during food processing, packaging, and storage, where frequent exposure to sublethal concentrations of sanitizing agents and industrial contaminants may facilitate the survival and persistence of more resistant strains.

Argudin et al. (2016) and Slifierz et al. (2015) both highlight the association between the use of metals and disinfectants in agricultural production settings and the resistance of *S. aureus* isolates. Argudin et al. (2016) found that livestock-associated *S. aureus* isolates, particularly CC398 strains, frequently carry metal resistance genes such as *arsA, cadD, copB*, and *czrC*, with significant differences between CC398 and non-CC398 strains. In line with these findings, our study also revealed that *S. aureus* isolates from ready-to-eat food products exhibited significantly higher resistance to both heavy metals and disinfectants compared to isolates from poultry and meat. Slifierz et al. (2015) demonstrated that in commercial swine herds, Zn in feed and frequent disinfection were significantly associated with Methicillin-Resistant *Staphylococcus aureus* (MRSA) shedding, with many MRSA isolates carrying the *czrC* Zn resistance gene and showing decreased susceptibility to Zn. Similarly, in our study, *S. aureus* isolates from ready-to-eat food products showed significantly higher resistance to Zn and Hg. These findings suggest that the use of heavy metals and disinfectants in agricultural and food production may facilitate the persistence and dissemination of antimicrobial-resistant *S. aureus*, including MRSA, raising concerns about potential zoonotic transmission.

The potential co-selection between heavy metal resistance and antimicrobial resistance genes should be considered, as plasmids or other mobile genetic elements often carry both, thereby enhancing the risk of resistance dissemination through the food chain (Balta et al. 2025). This interconnected resistance raises significant challenges for food safety control measures, emphasizing the need for stricter hygienic practices and enhanced surveillance strategies in ready-to-eat food production to mitigate the associated public health risks (Slifierz et al. 2015, Argudin et al. 2016).

The observed association between enterotoxin gene carriage and elevated resistance to antibiotics, disinfectants, and heavy metals suggests that virulence and antimicrobial resistance traits may be co-selected in *S. aureus* isolates. This finding is consistent with previous studies indicating that pathogenic strains often exhibit enhanced survival capabilities under environmental and chemical stressors (Argudin et al. 2016, Alkuraythi et al. 2023). One possible explanation is the presence of mobile genetic elements, such as plasmids or pathogenicity islands, which can carry both virulence factors and resistance genes, thereby facilitating the co-transfer and maintenance of these traits under selective pressure. Additionally, the food production environment may favor the persistence of highly virulent and resistant strains, particularly in niches where exposure to sublethal levels of disinfectants or heavy metals is frequent. The significantly higher resistance scores in enterotoxigenic isolates, as illustrated in Fig. 1, reinforce the need for integrated surveillance strategies that simultaneously monitor virulence and resistance markers. Such approaches are critical for risk assessment and for the development of effective control measures in both clinical and food-related settings.

Although some previous studies have suggested an inverse relationship between virulence and antimicrobial resistance, possibly due to metabolic trade-offs or regulatory mechanisms (Price and Boyd 2020, Ghssein and Ezzeddine 2022), the current findings clearly demonstrate a positive association. Specifically, enterotoxigenic isolates exhibited significantly higher resistance levels, in line with other reports indicating the co-selection and co-localization of virulence and resistance determinants, particularly in foodborne *S. aureus* strains (Argudin et al. 2016, Alkuraythi et al. 2023). This discrepancy with earlier studies may reflect differences in isolate origin, ecological niches, or environmental pressures specific to food production settings.

Overall, the data emphasize the importance of simultaneously monitoring both antimicrobial resistance and virulence profiles in *S. aureus* isolates, particularly in food safety and public health contexts (Alkuraythi et al. 2023, Chieffi et al. 2023). Further studies are warranted to explore the underlying genetic and regulatory mechanisms driving the observed co-occurrence of resistance and virulence traits.

From a food safety perspective, isolates from processed foods exhibited a higher frequency and broader range of resistance to heavy metals and disinfectants, suggesting greater exposure to environmental contaminants or selective pressures (Wales and Davies 2015, Chieffi et al. 2023). These foods often undergo longer and more complex processing chains, which may involve repeated exposure to sanitation agents, contact with contaminated equipment, and interactions with additives or preservatives (Vingadassalon et al. 2025). Such conditions may facilitate microbial adaptation and the development of resistance, thereby increasing the risk of resistant strains entering the food supply (Balta et al. 2025).

Furthermore, certain preservatives or additives in processed foods may exert selective pressure that contributes to co-selection mechanisms, favoring resistance not only to those compounds but also to antibiotics. This phenomenon is particularly relevant as resistance genes for metals, disinfectants, and antibiotics are frequently located on the same mobile genetic elements. Such physical linkage enables the co-selection and persistence of multidrug-resistant strains, even in the absence of direct antibiotic pressure (Slifierz et al. 2015, Balta et al. 2025).

Given the potential for cross-contamination during food production and the risk of foodborne outbreaks, the public health implications of these findings are considerable. The integration of antimicrobial resistance monitoring into routine food safety programs, alongside molecular epidemiological investigations, is essential to mitigate the risks associated with resistant *S. aureus* in the food supply chain (Argudin et al. 2016, Chieffi et al. 2023).

Future research should focus on expanding the isolate collection to further investigate resistance profiles across a broader range of food categories. Additionally, studying the effects of varying concentrations of disinfectants and heavy metals will provide deeper insights into resistance development. The mechanisms behind the transfer of resistance genes on mobile genetic elements, as well as the relationship between antibiotic and disinfectant resistance, should be explored in more detail. Molecular epidemiological studies can help trace the pathways of resistant isolates, contributing to more effective public health interventions. Finally, research on the impact of food processing and storage conditions on microbial resistance development could lead to the establishment of safer food production practices.

## Supplementary Material

fnaf109_Supplemental_File
