# Supplementary material for: Resistance beyond the plate: heavy metal, disinfectant, and antibiotic resistance in foodborne Staphylococcus aureus isolates
Source: FEMS Microbiol Lett. 2025 Oct 6;372:fnaf109. doi: 10.1093/femsle/fnaf109 (PMC12542497; doi:10.1093/femsle/fnaf109)
Supplement: fnaf109_Supplemental_File [file fnaf109_supplemental_file.docx]

**Supplementary Material**

Resistance profiles of *S. aureus* isolates from different food sources, including disinfectants, heavy metals, antibiotics, and toxin genes.

| Isolate | Category | Disinfectants | Heavy metals | Antibiotics | Toxin genes |
| --- | --- | --- | --- | --- | --- |
| 1a | Poultry | CC, BC | Zn, Hg, Co | - | - |
| 3a | Poultry | BC | Zn, Hg, Pb, Co | - | - |
| 7c | Poultry | CC, BC | Zn, Hg | P | - |
| 10a | Poultry | BC | Zn, Hg, Cd, Pb | P | - |
| 32a | Poultry | BC | Zn, Hg, Pb, Co | P | - |
| 34a | Poultry | BC, Tri | Zn, Hg, Cu, Co | P | - |
| 42a | Poultry | BC, | Zn, Hg, Cu, Pb, Co | CIP | - |
| 49a | Poultry | CC, BC | Zn, Hg, Cu, Cd, Pb, Co | P | - |
| 52a | Poultry | Tri | Zn, Hg, Cu, Co | CIP | - |
| 53a | Poultry | BC | Zn, Hg, Pb | P | - |
| 63a | Poultry |  | Zn, Hg, Cu, Co |  | SEE |
| 64c | Poultry | CC, BC | Zn, Hg, Cd, Pb | - | - |
| 74d | Poultry | CC, BC | Hg, Pb | CIP | - |
| 89b | Poultry | BC | Zn, Hg, Cu,Co | - | SEA, SEE |
| 105a | Poultry | BC |  | - | - |
| 107b | Poultry | CC, BC | Zn, Hg, Cu, Co | P, DA | - |
| 109a | Poultry | CC, BC | Zn, Cu, Cd, Pb, Co | - | - |
| 110a | Poultry | CC, BC | Zn, Hg, Cd, Pb | P | - |
| 111a | Poultry | CC, BC | Zn, Hg, Cu, Co | - | - |
| 113a | Poultry | BC | Zn, Hg, Cu, Co | CIP | - |
| 114a | Poultry | - | Zn, Hg, Cu, Co | - | SEA, SEE |
| 119a | Poultry | - | Zn, Hg | - | - |
| 8a | Meat | - | - | P, CIP | - |
| 19a | Meat | CC, BC | Zn, Hg, Cu, Cd, Co | P, CIP | - |
| 30a | Meat | - | Zn, Hg, Cu, | E, CIP, DA | - |
| 39b | Meat | - | Zn, Hg, Cu, Co | P, DA | - |
| 51a | Meat | - | - | P | - |
| 57c | Meat | - | Zn, Hg, Cd, Co | P | SEA |
| 66a | Meat | - | Zn, Hg, Cu, Co | - | - |
| 67b | Meat | BC | Zn, Hg, Cu, Co | - | - |
| 68a | Meat |  | Zn, Hg, Cu, Pb | P, STX, C | - |
| 86a | Meat | CC, BC | Zn, Cu | - | - |
| 92a | Meat |  | Zn, Hg, Pb, Co | P | SEA, SED, SEE |
| 101b | Meat | CC, BC | Zn, Hg, Pb | - | - |
| 104b | Meat | CC, BC | Zn, Hg, Pb | - | SEB, SEE |
| 167a | Meat | - | Zn, Hg, Cu, Cd, Pb, Co | - | - |
| 168b | Meat | BC | Zn, Hg, Cu, Cd, Co | - | - |
| 170a | Meat | BC | Zn, Hg, Cu, Cd | P, E, CIP, DA | - |
| 173b | Meat | CC, BC | Zn, Hg, Pb, Cd | - | - |
| 174a | Meat | - | Zn, Hg, Cu, Co | CIP, P | - |
| 175b | Meat | - | Zn, Hg, Cu, Co | - | - |
| 180a | Meat | BC | Zn, Hg, Cu, Co | - | - |
| 182a | Meat | - | Zn, Hg, Pb | - |  |
| 13d | Meat | CC, BC | Zn, Hg, Cu, Cd, Pb, Co | P, OX, FOX, E, CIP, DA | - |
| 15d | Processed | CC, BC | Zn, Hg, Cu, Cd, Pb, Co | P, OX, FOX, E, DA | - |
| 26d | Processed | - | Zn, Hg, Cu, Cd | P, OX, STX | - |
| 81a | Processed | - | Hg | P | - |
| 100d | Processed | CC, BC | Zn, Hg, Cu, Cd, Pb, Co | P, OX, FOX, E, DA | - |
| 122d | Processed | - | Zn, Co | P, OX, FOX, E, CIP, DA | - |
| 150d | Processed | - | Zn, Pb | FOX | SEC |
| 152a | Processed | BC | Zn, Hg, Cu, Cd, Co | FOX, CIP | SEC |
| 153b | Processed | CC, BC | Zn, Hg, Cu, Cd, Pb, Co | CIP | SEC |
| 155a | Processed | - | Co, Pb | P | - |
| 156a | Processed | CC, BC | Zn, Hg, Cu, Cd, Pb, Co | P, OX, FOX, E, DA | - |
| 158b | Processed | BC | Zn, Hg, Cu, Cd, Pb, Co | P, E, CIP | - |
| 160c | Processed | - | Zn, Hg, Cu, Co | FOX, CIP | - |
| 163d | Processed | BC | Zn, Cu, Cd, Co | FOX, CIP | - |
| 170a | Meat | CC, BC | Zn, Cd | P, OX, FOX, E, DA | - |
| 172c | Processed | CC, BC | Zn, Hg, Cu, Cd, Pb, Co | P, OX, FOX, E, DA | - |
| 177b | Processed | - | Zn, Hg, Cu, Cd, Pb, Co | P, E, CIP, DA | - |
| 180a | Meat | - | Zn, Hg, Cu, Cd | P, E | - |
| 191c | Processed | CC, BC | Zn, Hg, Cu, Cd, Pb, Co | CIP | - |
| 192b | Processed | - | Zn, Hg, Cd | P, E, CIP | - |

BC: Benzalkonium chloride; CC: Cetylpyridinium chloride; Co: Cobalt; Zn: Zinc; Cd: Cadmium; Cu: Copper; Hg: Mercury; Ni: Nickel; Pb: Lead; P: Penicillin; OX: Oxacillin; FOX: Cefoxitin; CN: Gentamicin; E: Erythromycin; CIP: Ciprofloxacin; DA: Clindamycin; SXT: Trimethoprim-sulfamethoxazole; C: Chloramphenicol; -: Not detected.
